# The Effect of Lamotrigine and Levetiracetam on TMS-Evoked EEG Responses Depends on Stimulation Intensity

**DOI:** 10.3389/fnins.2017.00585

**Published:** 2017-10-20

**Authors:** Isabella Premoli, Alyssa Costantini, Davide Rivolta, Andrea Biondi, Mark P. Richardson

**Affiliations:** ^1^Department of Basic and Clinical Neuroscience, Institute of Psychiatry, Psychology and Neuroscience, King's College London, London, United Kingdom; ^2^School of Psychology, University of East London, London, United Kingdom; ^3^Department of Education, Psychology and Communication, University of Bari Aldo Moro, Bari, Italy

**Keywords:** pharmaco-TMS-EEG, epilepsy, AED, transcranial magnetic stimulation, electroencephalography

## Abstract

The combination of transcranial magnetic stimulation and electroencephalography (TMS-EEG) has uncovered underlying mechanisms of two anti-epileptic medications: levetiracetam and lamotrigine. Despite their different mechanism of action, both drugs modulated TMS-evoked EEG potentials (TEPs) in a similar way. Since both medications increase resting motor threshold (RMT), the current aim was to examine the similarities and differences in post-drug TEPs, depending on whether stimulation intensity was adjusted to take account of post-drug RMT increase. The experiment followed a placebo controlled, double blind, crossover design, involving a single dose of either lamotrigine or levetiracetam. When a drug-induced increase of RMT occurred, post-drug measurements involved two blocks of stimulations, using unadjusted and adjusted stimulation intensity. A cluster based permutation analysis of differences in TEP amplitude between adjusted and unadjusted stimulation intensity showed that lamotrigine induced a stronger modulation of the N45 TEP component compared to levetiracetam. Results highlight the impact of adjusting stimulation intensity.

## Introduction

Levetiracetam and lamotrigine are two commonly prescribed anti-epileptic drugs (AEDs) (Nicholas et al., 2012). Lamotrigine blocks voltage-gated sodium channels and stabilizes their inactive state (Cheung et al., 1992), whilst levetiracetam inhibits the release of the excitatory neurotransmitter by binding to synaptic vesicle protein SV2A (Lynch et al., 2004). Electromyographic (EMG) responses to TMS indicate that lamotrigine and levetiracetam increase the resting motor threshold (RMT) (Ziemann et al., 1996), a TMS-EMG parameter which reflects neural membrane excitability and ion channel conductance (Ziemann et al., 1996; Solinas et al., 2008).

The combination of TMS with electroencephalography (TMS-EEG) demonstrates a greater potential to investigate effects of drugs *directly* at cortical level (Premoli et al., 2014a,b, 2016; Darmani et al., 2016). Single-pulses delivered over the motor area at threshold intensity (100% RMT) result in positive and negative deflections named TMS-evoked EEG potentials (TEP) which may provide insights into brain connectivity (Ilmoniemi et al., 1997) and neurotransmission (Premoli et al., 2014a). Recently, we used TMS-EEG to characterize the effects of single oral doses of lamotrigine and levetiracetam. Both AEDs increased the N45 and suppressed the P180. This study also showed the expected finding that these AEDs both increase RMT (Premoli et al., 2016).

In studies examining the effects of drugs, it is conventional to obtain TMS measurements in two sessions, pre-drug and post-drug intake. Conventionally, TMS intensity is calibrated against RMT in the pre-drug session and not adjusted in the post-drug session, even if RMT has changed. Therefore, the absolute stimulation intensity is the same between sessions but the relative intensity (relative to RMT) may vary between sessions. It could be equally valid to adjust RMT between sessions so that the relative stimulation intensity is the same between sessions but the absolute intensity may vary.

Sitting in the cognitive domain of TMS literature, the non-linear effects of stimulation intensity on behavior are well-known. A well-documented review provides details of how stimulation parameters (i.e., intensity), brain state dependency, and task characteristics impact cortical excitability and therefore behavioral responses (Miniussi et al., 2013). For instance, it has been shown that the impact of mental imagery contrast on phosphene perception is strictly dependent on TMS intensity (Cattaneo et al., 2011), which therefore affects TMS assessment of visual cortical excitability. In line with this view, a previous work demonstrated that, during visually evoked neural activity in anaesthetized and paralyzed cats, low intensity TMS has facilitatory effects on neural firing, whereas high intensity TMS produces an opposite behavior (Moliadze et al., 2003).

A similar problem may arise also in non-pharmacological interventions, that by adopting neuromodulation techniques such as repetitive TMS (i.e., rTMS), can induce RMT changes (Muellbacher et al., 2000; Fitzgerald et al., 2002, 2006). It is therefore a relevant issue to monitor and consider RMT changes before and after pharmacological and non-pharmacological stimulation protocols.

In our recent study, we presented data in which TMS stimulation intensity was not adjusted in the post-drug condition (Premoli et al., 2016). However, we also collected data in which TMS intensity was adjusted according to RMT in the post-drug session. Here, we examine whether observed effects on TEPs following drug intake are partly dependent on adjustment of post-drug TMS intensity to take account of the change in RMT.

## Materials and methods

### Experimental procedure

Fifteen male subjects aged 19–34 years (mean age ± *SD*, 25.2 ± 4.62 years) gave written informed consent before enrolment in this study. One subject only was not able to complete the TMS-EEG recording after the intake of lamotrigine; hence the total number of subjects for this condition is fourteen. The study was performed according to the Declaration of Helsinki and approved by King's College London Research Ethics Committee.

Specific subject information, TMS-EMG/-EEG equipment, data processing, and analysis protocols were described in our previous work (Premoli et al., 2016). The experiment followed a pseudo-randomized, placebo controlled, double-blinded crossover study design in which all subjects participated in three separate sessions, at intervals of 1 week, to receive a single oral dose of levetiracetam (3,000 mg) or lamotrigine (300 mg) or placebo (Premoli et al., 2016). During each session, data were collected before and 120 min after drug intake. When a drug-induced increase of RMT occurred, the post-drug measurements involved two blocks of stimulations. In a randomized order, TEPs were recorded at an unadjusted (100% pre-drug RMT = RMT1) and adjusted (100% post-drug RMT = RMT2) stimulation intensity. In our previous work we reported only the RMT1 condition (Premoli et al., 2016).

Following the relative frequency method (Groppa et al., 2012), the RMT was defined as the lowest stimulus intensity sufficient to elicit an MEP of >50 μV peak-to-peak amplitude in at least 5 out of 10 trials whilst the first dorsal interosseous (FDI) was relaxed. Despite the common knowledge that 100% RMT can elicit no or only miniature MEPs, we cannot completely exclude that TEPs were not contaminated by somatosensory afferent signals from muscle twitches, as assessed by recent studies (Fecchio et al., 2017; Petrichella et al., 2017; Premoli et al., 2017).

The hotspot position and the edge of each coil's wing were marked on top of the EEG cap using a felt tip pen. Further, the same experimenter conducted all the TMS-EEG sessions for each participant and study session. Coil position and orientation relative to the marked position were carefully monitored by the experimenter throughout stimulation and corrected if necessary (i.e., if the participant moved) (Premoli et al., 2016).

Details for the multistep procedure of TMS-EEG data preprocessing are provided in our previous work (Premoli et al., 2016). Independent component analysis (FastICA), as implemented in FieldTrip following an approach based on Korhonen et al. (2011) (http://www.fieldtriptoolbox.org/tutorial/tms-eeg), was applied to remove TMS-related artifacts (i.e., cranial muscle response, recharging of capacitors, and related exponential decay artifacts; Korhonen et al., 2011; Rogasch et al., 2014; Herring et al., 2015) as well as further muscle and ocular activity. The criteria for components removal were if their spatio-temporal profile indicated the activation of temporal muscles by a characteristic sinusoidal waveform post-TMS (with opposite sign) over frontotemporal sites close to the temporal muscle (Veniero et al., 2013; Rogasch et al., 2014; Herring et al., 2015; Premoli et al., 2017). To note, in line with the recent work from Casula and colleagues, FastICA can only attenuate decay artifacts (Casula et al., 2017).

Five TEP components in accordance with the literature (Premoli et al., 2014a, 2016) were studied: P25 [time of interest TOI (15–35 ms)], N45 (35–65 ms), P70 (65–90 ms), N100 (90–145 ms), and P180 (145–300 ms). TOIs were chosen on the basis of the grand-averaged TEPs and kept identical during the analysis of pre-drug and post-drug measurements and across conditions. To analyze drug-induced modulation of TEPs, we selected a region of interest (ROI) composed of 12 channels over and around the stimulation site (left M1) and the corresponding contralateral site (FC1, FC3, FC5, C1, C3, C5, CP1, CP3, CP5, P5, P3, P1, FC2, FC4, FC6, C2, C4, C6, CP2, CP4, CP6, P2, P4, and P6).

### Statistics

Multiple dependent sample *t-test* comparisons were separately applied for each TOI in all the electrodes within the indicated ROI to (i) test TEP amplitude modulations induced by lamotrigine and levetiracetam (post-drug with RMT1 vs. pre-drug; post-drug with RMT2 vs. pre-drug), to (ii) analyse the effect of different stimulation intensities (post-drug with RMT2 vs. post-drug with RMT1), and to (iii) understand the interaction of these two factors [levetiracetam (post-RMT2 *minus* post-RMT1) vs. lamotrigine (post-RMT2 *minus* post-RMT1)]. To correct for multiple comparisons (i.e., electrodes, time points), and in line with our previous work (Premoli et al., 2016), we conducted a non-parametric cluster-based permutation analysis as implemented in FieldTrip (Maris and Oostenveld, 2007).

## Results

### Effects of AEDs and increased stimulation intensities on TEPs

**Figure 1:**
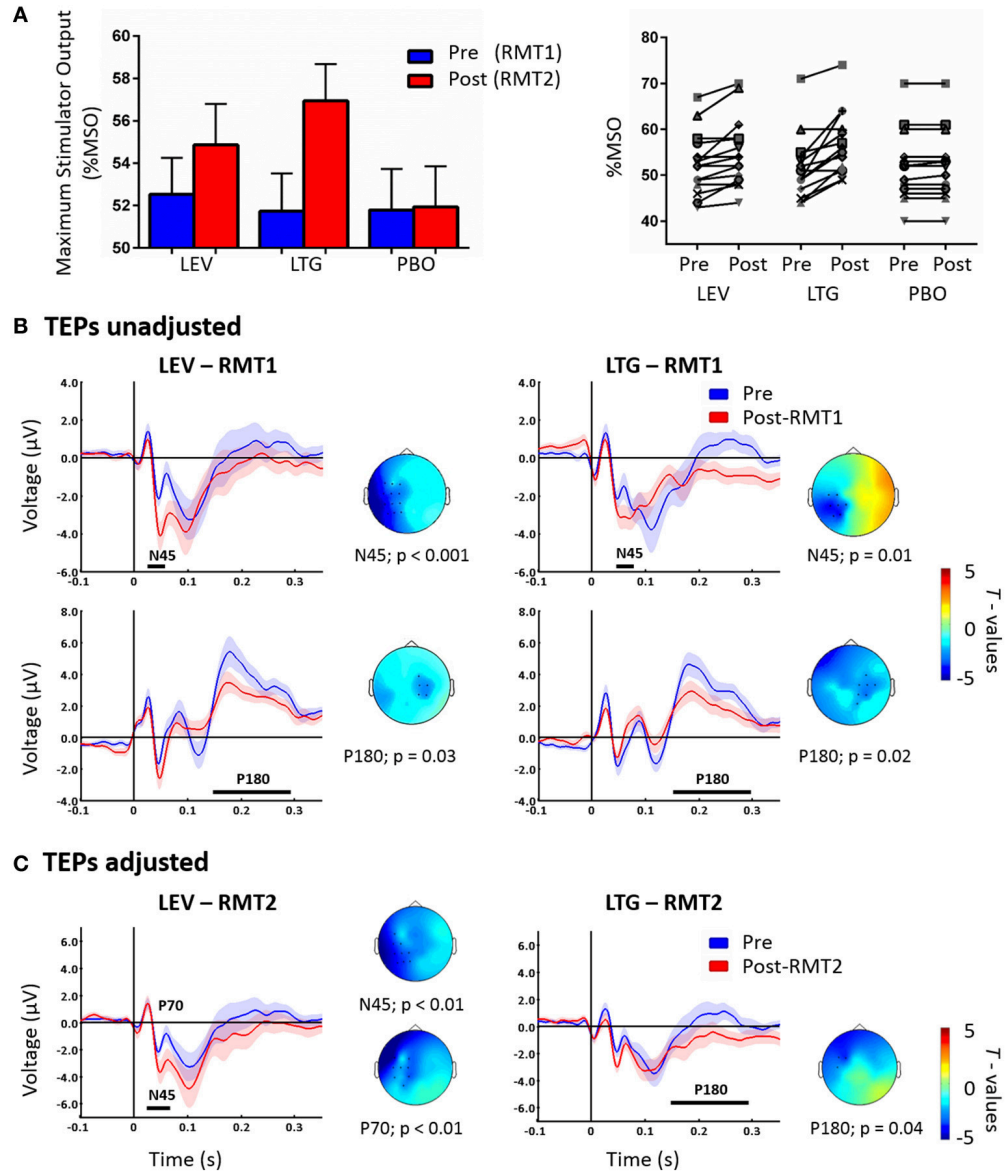
Left part of **(A)** shows RMT-values given as % of Maximum Stimulator Output (MSO) pre (blue = RMT1) and post (red = RMT2) the intake of levetiracetam (LEV), lamotrigine (LTG), and placebo (PBO). The right part shows individual RMT values (expressed as % MSO) before and after the intake of each drug condition. **(B)** TEPs recorded pre (blue) and post-RMT1 (red; unadjusted intensity) intake of levetiracetam (LEV, left) and lamotrigine (LTG, right). Levetiracetam and lamotrigine increased the N45 over the left hemisphere and decreases the P180 component over channels contralateral to the stimulated left M1. Adapted from Premoli et al. (2016). **(C)** TEPs recorded pre (blue) and post-RMT2 (red; adjusted intensity) intake of levetiracetam (LEV, left) and lamotrigine (LTG, right). Levetiracetam increased the N45 and decreased the P70, while lamotrigine suppressed the P180. Effects are observed over the stimulated left hemisphere. Black asterisks underneath represent significant drug-induced changes in TEPs. Shades indicate ± 1 SEM. T-statistic maps of the TEP amplitude post-drug vs. pre-drug differences are shown for each comparison. Blue represents increase in negativity or reduced positivity. Each TEP plot shows the grand-average across significant channels which are indicated by black dots in the t-statistic maps.

Lamotrigine and levetiracetam produced a significant RMT increase as expected (Solinas et al., 2008; Premoli et al., 2016) (Figure 1A, left panel); these data are explained in details in our previous work (Premoli et al., 2016). Levetiracetam and lamotrigine individual changes of RMT values showed an increase in 11 and 13 subjects, respectively (Figure 1A, right panel). We here present the results reported previously, comparing post-drug with RMT1 (i.e., unadjusted) intensities against pre-drug conditions (Premoli et al., 2016). Figure 1B shows that both AEDs increased the amplitude of the N45 over channels ipsilateral to the stimulated left M1 and suppressed the P180 component over contralateral sites.

The cluster analysis did not show significant effects (*p* > 0.05) between the pre-drug conditions showing high reproducibility across sessions (Premoli et al., 2016). Cluster-based permutation analysis was applied between post-RMT2 and pre-drug conditions to test the effect of AEDs on TEPs recorded with adjusted stimulation intensity. Levetiracetam increased the N45 amplitude (*n* = 15, *p* = 0.007) and suppressed the P70 amplitude (*n* = 15, *p* < 0.01) on channels close to the stimulation site. Levetiracetam showed a trend for the suppression of the P180 (*p* = 0.057). In contrast, lamotrigine suppressed only the P180 amplitude (*n* = 15, *p* = 0.04) over ipsilateral channels (Figure 1C). Finally, no significant differences were observed after placebo (*p* > 0.05).

### Effect of stimulation intensities

**Figure 2:**
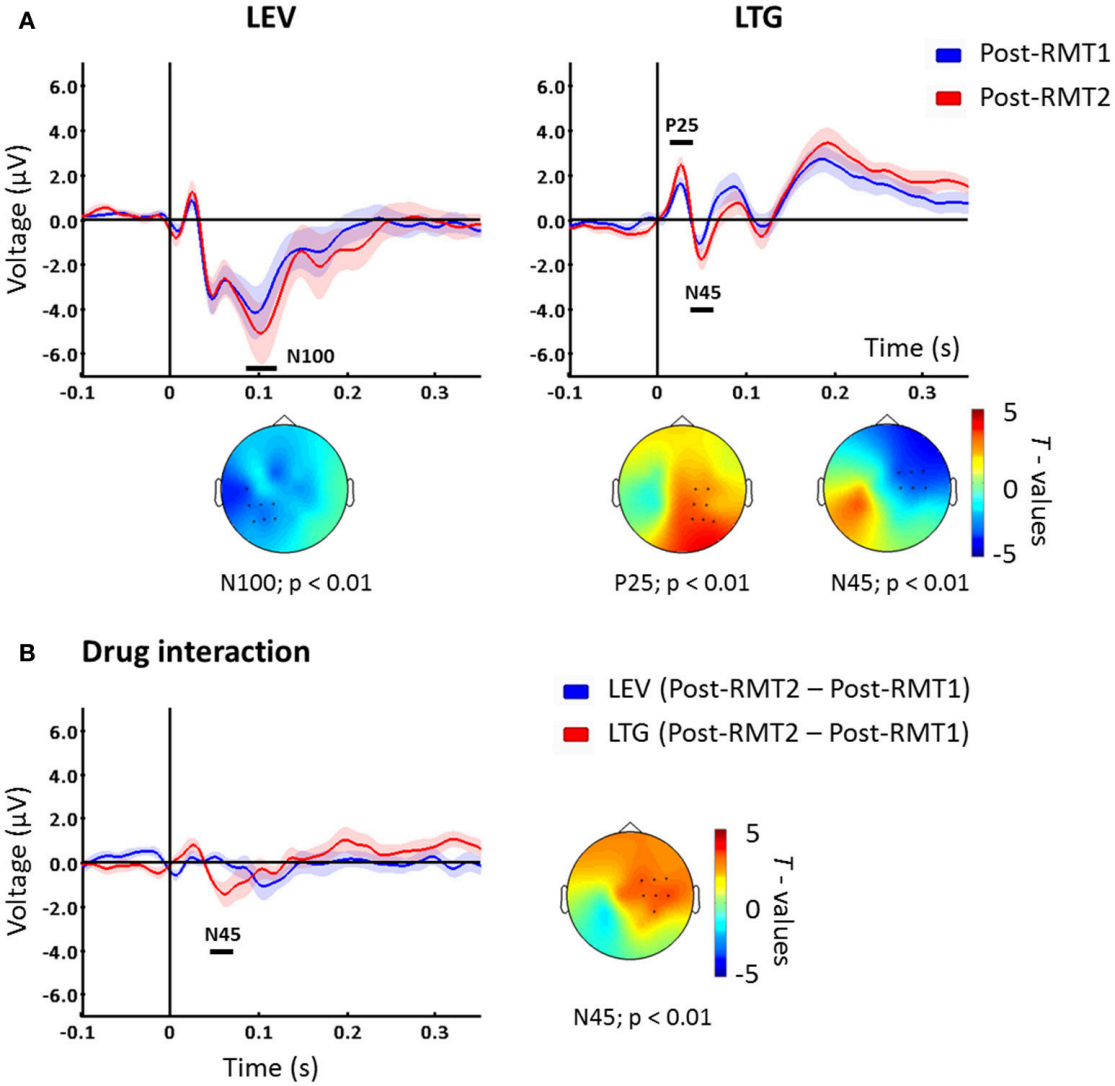
**(A)** TEPs measured post drug conditions with unadjusted (Post-drug with RMT1, blue) and adjusted (Post-drug with RMT2, red) intensity. Levetiracetam (left) increased the N100 potential over the left hemisphere, while lamotrigine increased P25 and N45 peaks on channels contralateral to the stimulated left M1. T-statistic maps of the TEP differences are shown. Blue represents increase in negativity (i.e., N100 and N45) and red increased positivity (i.e., P25) **(B)** Difference between Post-drug with RMT2 and Post-drug with RMT1 for levetiracetam (blue) and lamotrigine (red) which showed a significant difference corresponding with the N45 potential latency over contralateral sites as indicated in the t-statistic map. Black bars underneath each curve represent significant changes. Shades indicate ± 1 SEM. Each plot shows the grand-average across significant channels which are indicated by black dots in the *t*-statistic maps.

To investigate the effects of the increased stimulation intensity, we compared post-drug using RMT2 vs. post-drug using RMT1. For levetiracetam, TEPs resulting from adjusted stimulation intensity (RMT2) compared to unadjusted RMT1 showed increased N100 over channels close to the stimulation site (*p* < 0.01; Figure 2A). In contrast, post-lamotrigine RMT2 compared to an unadjusted RMT1 demonstrated increased P25 amplitude (*p* < 0.01; Figure 2A) and augmented N45 amplitude (*p* < 0.01; Figure 2A) over contralateral areas. In the placebo condition there were no significant differences (*p* > 0.05).

### Interaction between stimulation intensity and AEDs effects

Finally, we explored the interaction between stimulation intensity and the different medications. To address this, we compared intensity-induced changes in TEPs (post-RMT2 *minus* post-RMT1 intake) between drug conditions. The comparison showed that, when stimulating at higher intensities, lamotrigine induced a greater modulation of the N45 over contralateral channels (*p* < 0.01, Figure 2B) compared to levetiracetam.

## Discussion

We have recently demonstrated that, despite the different mechanism of action that lamotrigine and levetiracetam exert at the molecular level, both AEDs impact the TMS-EEG response in a similar way. The modulation induced by lamotrigine and levetiracetam on TEPs recorded with unadjusted stimulation intensity consisted of an increased amplitude of the N45 and a suppressed P180 (Premoli et al., 2016). We here sought to investigate the impact of increased stimulation intensity, adjusted because of post-drug increased RMT, when assessing AEDs effects on TMS-evoked EEG responses. We found that the N100 component was altered by the higher intensity of the RMT2 condition compared to RMT1 for levetiracetam, whereas P25 and N45 were altered in a specific way in the RMT2 condition compared to RMT1 for lamotrigine. When we compared the effect of RMT2 vs. RMT1 between drugs, we found that the N45 potential was affected. Therefore, the effect of drugs on TEPs differs depending on whether *absolute* stimulation intensity or *relative* stimulation intensity is held constant between pre- and post-drug conditions.

TMS stimulation at higher intensity can produce a stronger neuronal activity measured with EEG. It has been shown that the amplitudes of N45 depended on intensity in a non-linear manner, while the amplitude of the N100 and P180 components was rather linear. Further, these changes occurred in the same cortical structures independently of stimulus intensities (Komssi et al., 2004). Our results suggest that when stimulation intensity is adjusted to take account of increased post-drug RMT, the effects of lamotrigine and levetiracetam on TEPs differ compared to the unadjusted condition. Comparison between stimulation intensities for levetiracetam showed a suppression of the difference curve (post-RMT2 minus post-RMT1) toward the baseline, thus indicating that levetiracetam exerts the same modulation independently of the stimulation intensity for each potential. Conversely, at adjusted RMT values, lamotrigine induces a larger N45 potential, a component which is associated with GABA-A receptor (GABAAR) mediated inhibitory neurotransmission (Premoli et al., 2014a,b; Darmani et al., 2016). This result raises two crucial questions: why does the modulation affect specifically the N45 component, which has been related to GABAAR activity (Premoli et al., 2014a,b; Darmani et al., 2016)? Why does lamotrigine show a greater modulation at adjusted (i.e., increased) stimulation intensities?

There is little evidence at present to answer these questions. However, lamotrigine is often used as a mood stabilizer, in addition to being a broad spectrum anti-convulsant. This could be indicative of additional actions in the brain other than being solely thought of as a traditional sodium-channel blocker. For instance, lamotrigine has also been suggested to have an indirect effect on GABAAR mediated neurotransmission (Cunningham and Jones, 2000) which may explain the modulation of the N45 potential, a potential marker for GABAAR activity (Premoli et al., 2014a,b; Darmani et al., 2016). In addition, the channels which showed N45 significant changes were located contralateral to the stimulated site, in line with the topographical modulation induced by positive modulators of GABAAR (Premoli et al., 2014a). Another key of interpretation may be a global shift of the excitation/inhibition balance toward the latter, a common mechanism of action for AEDs (Greenhill and Jones, 2010). Future studies should assess whether lamotrigine-responsiveness (i.e., seizure freedom) in patients with epilepsy is associated to the N45 modulation, a candidate biological measure of treatment outcome.

Of relevance to the second question, we refer to a previous TMS/fMRI study which investigated the effects of lamotrigine after threshold and suprathreshold TMS stimulation. In line with our finding of a greater modulation at RMT2 levels, results showed a reduction of TMS-induced BOLD activation of the motor area after lamotrigine administration, with even stronger effects when TMS was applied at 120% RMT compared to 100% RMT (Li et al., 2004).

In the non-invasive brain stimulation field, the choice of stimulation parameters such as intensity is of high relevance and it is crucial for the outcome of investigational and therapeutic studies (Fitzgerald et al., 2006). It becomes even a more relevant challenge when targeting non-motor sites as it is difficult to disentangle excitability changes of the stimulated neural population. When designing experiments which include post-drug or post-intervention RMT measurements, it should be considered that RMT assessment can be time consuming and may affect the investigation of short-term after effect. In this particular study, the effects of the two medications on cortical excitability lie in a wide time window appropriate for post-drug investigations. It was shown that the effects of lamotrigine (Tergau et al., 2003) and levetiracetam (Epstein et al., 2008) on RMT remained significantly elevated from 2 to 8 and 1 to 24 h post-dose, respectively.

To conclude, we show that the increased stimulation intensity determined a different mechanistic profile evaluated with TEPs. Our results indicate that in future pharmaco-TMS-EEG experiments a range of stimulation intensities should be used in both pre- and post-drug conditions, in order to enable comparison of TEPs between conditions using relative vs. absolute stimulation intensity.

## Author contributions

IP and MR contributed to the design and write-up. IP, AC, DR, and AB contributed to recordings, analyses, and write-up of the study.

### Conflict of interest statement

The authors declare that the research was conducted in the absence of any commercial or financial relationships that could be construed as a potential conflict of interest.
